# Primate abnormal spindle-like microcephaly-associated knockout causes severe microcephaly and oligodendrocyte loss in the brain

**DOI:** 10.1093/procel/pwaf097

**Published:** 2025-11-10

**Authors:** Dajian He, Mingtian Pan, Fengwei Sun, Liang Jiang, Qintian Guo, Peng Wu, Shihua Li, Weili Yang, Zhuchi Tu, Xiao-Jiang Li, Xiangyu Guo

**Affiliations:** Guangdong Provincial Key Laboratory of Non-human Primate Research, GHM Institute of CNS Regeneration, Jinan University, Guangzhou 510632, China; Lingang Laboratory, Shanghai 20031, China; Guangdong Provincial Key Laboratory of Non-human Primate Research, GHM Institute of CNS Regeneration, Jinan University, Guangzhou 510632, China; Lingang Laboratory, Shanghai 20031, China; Guangdong Provincial Key Laboratory of Non-human Primate Research, GHM Institute of CNS Regeneration, Jinan University, Guangzhou 510632, China; Guangdong Provincial Key Laboratory of Non-human Primate Research, GHM Institute of CNS Regeneration, Jinan University, Guangzhou 510632, China; Guangdong Provincial Key Laboratory of Non-human Primate Research, GHM Institute of CNS Regeneration, Jinan University, Guangzhou 510632, China; Guangdong Provincial Key Laboratory of Non-human Primate Research, GHM Institute of CNS Regeneration, Jinan University, Guangzhou 510632, China; Guangdong Provincial Key Laboratory of Non-human Primate Research, GHM Institute of CNS Regeneration, Jinan University, Guangzhou 510632, China; Guangdong Provincial Key Laboratory of Non-human Primate Research, GHM Institute of CNS Regeneration, Jinan University, Guangzhou 510632, China; Guangdong Provincial Key Laboratory of Non-human Primate Research, GHM Institute of CNS Regeneration, Jinan University, Guangzhou 510632, China; Guangdong Provincial Key Laboratory of Non-human Primate Research, GHM Institute of CNS Regeneration, Jinan University, Guangzhou 510632, China; Lingang Laboratory, Shanghai 20031, China; Guangdong Provincial Key Laboratory of Non-human Primate Research, GHM Institute of CNS Regeneration, Jinan University, Guangzhou 510632, China

**Dear Editor,**

Although brain development and expansion critically depend on neurogenesis and gliogenesis, the mechanisms driving cerebral cortical gyrification and enlargement in primates remain incompletely understood (Akula et al., 2023). Among the many genes linked to microcephaly primary hereditary (MCPH), mutations in the abnormal spindle-like microcephaly-associated (*ASPM*) gene are the most common cause (Bond et al., 2002; Létard et al., 2018). *ASPM* is predominantly expressed in the progenitor cells of the developing brain, specifically in the ventricular and subventricular zones of the cerebral cortex, where its expression peaks during neurogenesis and declines postnatally (Bond et al., 2002). This developmentally regulated expression underscores its critical role in the expansion of the cerebral cortex. However, the pathological consequences of *ASPM* mutation in non-human primates remain unexplored. Given the lack of cerebral cortical gyrification in rodents as well as other small animals and the evolutionarily divergent functions of *ASPM* (Montgomery and Mundy, 2014), it is important to use larger animal models that exhibit gyrification to investigate *ASPM* function.

We used CRISPR/Cas9 to target exon 3 and exon 9 of the monkey *ASPM* gene to generate a complete knockout (Fig. 1A; Table S1). *ASPM* gRNA and Cas9 mRNA were injected into fertilized cynomolgus monkey eggs, which were then transferred to surrogate females, resulting in six live births (Fig. 1B). Using placental tissue from newborns or brain tissue from abortions, we verified *ASPM* editing by using a T7E1 assay for exon 3 and a *Bsm*AI digest assay for exon 9, as the exon 9 target sequence contains a *Bsm*AI recognition site (Fig. S1A). Of the six newborns, one (*ASPM* KO, male) displayed an obviously smaller head and body compared to an age-matched male control monkey born on the same day (Fig. 1C). The other five newborns showed normal development and activity. Analysis of blood samples indicated these monkeys retained a wild-type *ASPM* allele. Their lack of an abnormal phenotype suggests the persistence of functional *ASPM* in the brain, consistent with a heterozygous or mosaic state. For this reason, we concentrated further investigation on the *ASPM* KO monkey, which is putatively homozygous for the mutation, and its genetically matched control.

**Figure 1.**
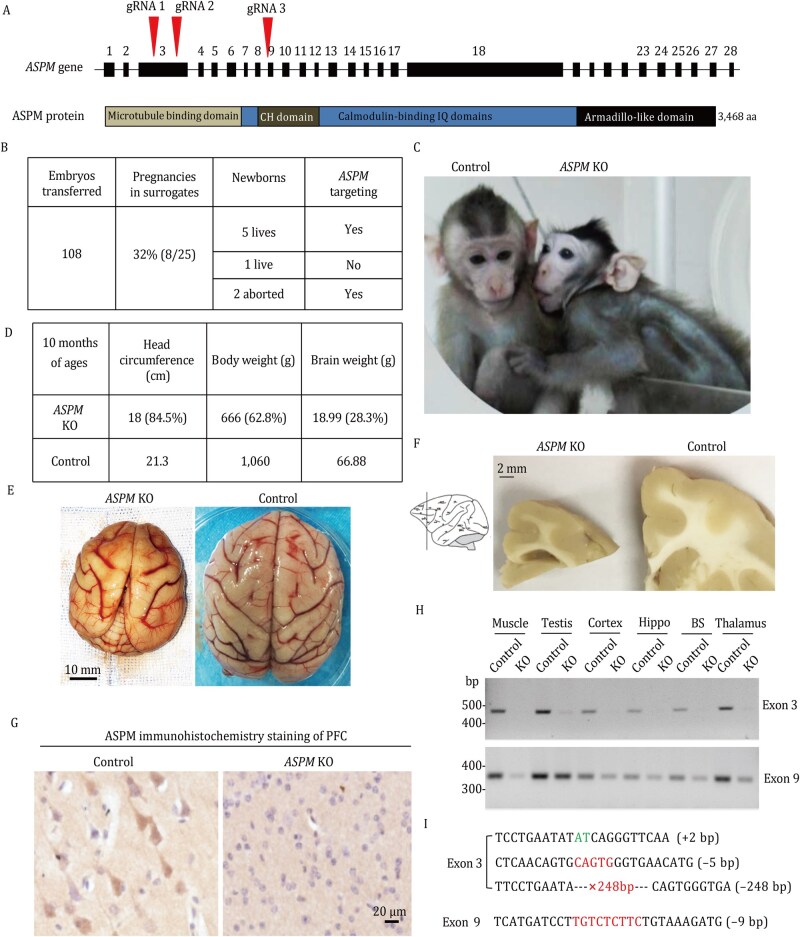
**Generation and validation of *ASPM* knockout cynomolgus monkeys**. (A) Schematic of the gRNA target sites in exons 3 and 9 of the cynomolgus monkey *ASPM* gene. (B) Six live monkeys were born following the transfer of 108 embryos that had been injected with *ASPM*-targeting gRNAs and Cas9 mRNA. (C) Photographs of an 8-month-old *ASPM* knockout (KO) monkey and an age-matched wild-type (WT) control. (D) Body weight and head circumference measurements of the *ASPM* KO monkey and the control at 3 months of age. (E and F) Reduced brain volume (E) and brain cortical size (F) in the *ASPM* KO monkey compared to the control monkey. The diagram on the left indicates the sectioning plane. (G) Immunostaining for ASPM protein in the prefrontal cortex revealing the absence of *ASPM* in the *ASPM* KO monkey. (H) PCR genotyping shows the absence of exon 3 and reduced amplification of exon 9 in various tissues from the *ASPM* KO monkey. Primer pairs: Exon3-3F-1/Exon3-3R (exon 3) and Exon9-9F-1/Exon9-9R (exon 9). (I) DNA sequencing reveals frameshift mutations and a large deletion (−248 bp) in exon 3 and a 9 bp deletion in exon 9.

The *ASPM* KO monkey consistently exhibited a smaller body and head at 3, 6, and 9 months of age (Fig. S1B). This mutant also showed reduced movement and exploratory activity compared to the control (Fig. S1C and Video S1). The *ASPM* KO monkey unfortunately died from pneumonia at 9 months of age, which prevented the longitudinal analysis of its behavioral phenotypes. Analysis of peripheral tissues from the deceased *ASPM* KO monkey revealed pulmonary edema, with no significant morphological changes in the liver, heart, or kidney (Fig. S2). Measurements of head circumference, body weight, and brain weight revealed that the whole brain weight of the *ASPM* KO monkey was 28.4% of the control, while its body weight was 62.8% of the control (Fig. 1D and 1E). Sectioning of the prefrontal cortex clearly showed a much smaller cortex in the *ASPM* KO monkey compared to the control (Fig. 1F). Immunocytochemistry of the prefrontal cortex also confirmed the elimination of ASPM protein expression in the KO monkey (Fig. 1G). Using PCR with primers specific for the targeted sequences in exons 3 and 9, we found that exon 3 was completely disrupted while exon 9 was partially mutated in different tissues of the *ASPM* KO monkey (Fig. 1H). DNA sequencing revealed a 9 bp deletion in exon 9 and three distinct frameshift mutations in exon 3—a 2 bp insertion, a 5 bp deletion, and a 246 bp deletion—all predicted to cause premature termination of the ASPM protein (Fig. 1I).

Although the cortical gray matter was much thinner in the *ASPM* KO monkey (Figs. 2A and S31), the six cortical layers remained intact (Fig. 2B). However, in the thinner cortical layers of the *ASPM* KO monkey, the density of NeuN-positive cells appeared increased, and fewer cells exhibited long neurites compared to the control (Fig. 2C). Western blot of ASPM KO brain tissue showed that DCX, a marker of newborn neurons, was elevated (Fig. 2D), suggesting that *ASPM* KO neurons are less mature. Single-cell RNA-seq analysis of the non-human primate brains indicated that *ASPM* is expressed in oligodendrocytes (Han et al., 2022; Fig. S3B). Notably, oligodendrocyte density was remarkably reduced in the *ASPM* KO monkey (Fig. 2E), while astrocytes and microglia were unaltered (Fig. S4). Quantification confirmed a decrease in oligodendrocyte density and an increase in neuronal density (Fig. 2F). Consistent with the immunocytochemical staining, myelin proteins (MBP, Olig2, and MOG), which are produced by oligodendrocytes, were also decreased, as shown by Western blot (Fig. 2G) and quantification of their ratios to vinculin (Fig. 2H). In accordance with the important role of myelination in maintaining neuronal synapses (Wang et al., 2018), Western blot also demonstrated reductions in synaptic proteins (PSD95, synaptophysin, SNAP25, and MAP2) in the *ASPM* KO monkey (Fig. 2I).

**Figure 2.**
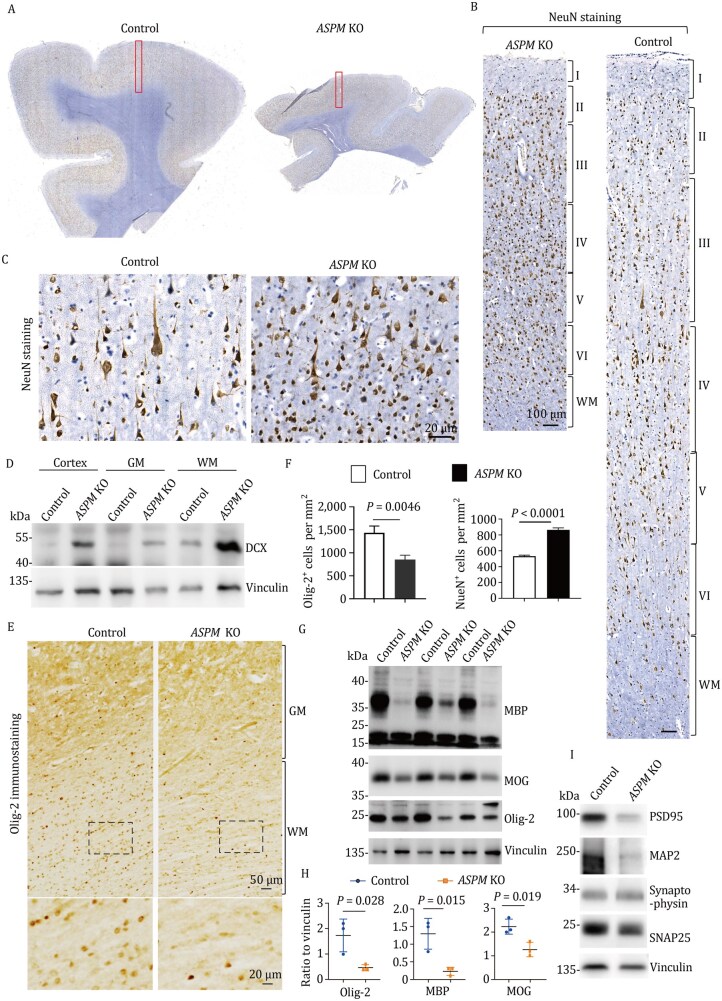
***ASPM* deficiency leads to impaired cortical development and reduced oligodendrocyte numbers**. (A and B) Comparative view of the prefrontal cortex (PFC) size in *ASPM* KO and control monkeys (A). The boxed region was sectioned and stained for the neuronal marker NeuN (B). (C) High-magnification images of NeuN staining show increased cell density and altered neuronal morphology in the *ASPM* KO PFC. (D) Western blot analysis shows an increased level of Doublecortin (DCX), an immature neuron marker, in the *ASPM* KO brain, indicating impaired neuronal maturation. (E) Immunostaining for the oligodendrocyte lineage marker Olig2 shows a reduction in oligodendrocytes in the white matter of the *ASPM* KO monkey. (F) Quantification of Olig2-positive and NeuN-positive cells in the PFC. Data are presented as mean cell density (cells/mm^2^) ± SEM (*n *= 4 sections per monkey, with at least 4 images analyzed per section). The data were analyzed by Student’s *t*-test. (G) Western blot analysis of myelin-related proteins (MBP, MOG) and the oligodendrocyte marker Olig2 in the PFC of *ASPM* KO and control monkeys. Vinculin serves as a loading control. (H) Densitometric quantification of the western blots shown in (G), normalized to Vinculin. Data are presented as mean fold change ± SEM from three independent blots. The data were analyzed by Student’s *t*-test. (I) Western blot analysis shows a reduction in synaptic markers (PSD95, MAP2, Synaptophysin, and SNAP25) in the *ASPM* KO PFC.

While *Aspm* KO mice display only mild microcephaly (4%–12% reduction) (Fujimori et al., 2014; Jayaraman et al., 2016; Pulvers et al., 2010; Williams et al., 2015) and humans show a > 50% reduction in cortical volume (Passemard et al. 2016), the *ASPM* KO monkey exhibits a remarkably severe phenotype with over 70% reduction in brain weight. This finding underscores the critical importance of large animal models with a gyrated cerebral cortex for modeling *ASPM* function. The phenotype was also more severe than in the *Aspm* KO ferret, which shows a 20%–40% decrease in brain weight (Johnson et al., 2018). We compared *ASPM* mutations and associated phenotypes across animal models. *ASPM* undergoes alternative splicing, resulting in different isoforms in humans and mice (Fig. S5). Human *ASPM* has two additional truncated isoforms that lack exons 4–17 and are absent in mice (Fig. S5). In the *Aspm* KO ferret model, TALEN-mediated targeting of exon 15 created a single exon mutation that also occurs in humans (Johnson et al., 2018). While patient-derived brain organoids with *ASPM* mutations (exons 18 and 25) revealed a neurogenesis defect (Li et al., 2017), examining oligodendrocyte-related phenotypes in organoids remains challenging. In our *ASPM* KO monkeys, however, two exons (3 and 9) were targeted. Targeting both exon 3 and exon 9 is likely to eliminate the expression of all protein isoforms and may therefore result in loss of oligodendrocytes in the developing brain and more severe microcephaly.

The complete loss of *ASPM* function, resulting from multi-exon targeting, may underlie the selective reduction of oligodendrocytes—a phenotype not reported in other *ASPM* KO animals. Gliogenesis in the outer subventricular zone, a brain region absent in rodents, plays a critical role in the expansion and gyrification of the primate cerebral cortex (Rash et al., 2019). Recent studies of human postnatal cortical development have revealed that myelination, a key function of oligodendrocytes, is critical for infant cortical development and correlates with cortical thickness (Natu et al., 2019). A striking observation in our study was the severe deficit of oligodendrocytes in the *ASPM* KO monkey, which was not accompanied by significant abnormalities in astrocytes or microglia. This selective loss suggests that *ASPM* plays a critical and specific role in the generation of oligodendrocytes during early brain development. As a centrosomal protein essential for mitotic spindle formation, the loss of *ASPM* likely disrupts the division and proliferation of oligodendrocyte progenitor cells. Since oligodendrocyte generation is critical for cortical expansion in primates, this specific deficit could explain the more severe microcephaly observed in *ASPM*-knockout primates compared to rodents.

A limitation of our study is that only one *ASPM* KO monkey was successfully generated among six live births, which limits the statistical power and generalizability of the findings. The rarity of biallelic knockout animals in non-human primates (NHPs) is a common challenge due to the inefficiencies of CRISPR/Cas9 editing and ethical constraints. Furthermore, the *ASPM* KO monkey died at 9 months of age from pneumonia, preventing long-term observation of developmental trajectories, behavioral outcomes, or potential compensatory mechanisms. Despite these limitations, our study provides compelling evidence for *ASPM*’s role in primate brain development and highlights the value of NHP models for studying neurodevelopmental disorders. Future research should explore the molecular pathways through which *ASPM* regulates oligodendrocyte development and investigate potential therapeutic strategies to mitigate the effects of *ASPM* deficiency.

## Supplementary Material

pwaf097_Supplementary_Data

## Supplemental data

All data are incorporated into the article and its online supplementary material.

## Footnotes

The authors thank Yuanxi Biotech Inc. Guangzhou for providing animal facility and animal care. We thank Xiao Zhang at BGI Research for single cell RNAseq data analysis and Miss. Haiying Wang of Indica Labs HALO team for supporting IHC image analysis. This work was supported by National Key Research and Development Program of China (2021YFF0702201, 2021YFF0702204), The National Natural Science Foundation of China (81873736, 82394422, 82371874, 32170981, U24A6013, 82271902, 32370567, 32370567), Department of Science and Technology of Guangdong Province (2021ZT09Y007, 2020B121201006, 2022A1515012651), Guangdong Basic and Applied Basic Research Foundation (2023B1515020031).

X. L. and X. G. conceived the project idea and wrote the paper. Z. T., W. Y., and X. G. established the animal model. D. H. M.P., F. S., L. J., Q. G., and P.W. analyzed *ASPM* KO monkey and performed the histological experiments. S.L. and W. Y. provided advice and edited the manuscript.

Dajian He, Mingtian Pan, Fengwei Sun, Liang Jiang, Qintian Guo, Peng Wu, Shihua Li, Weili Yang, Zhuchi Tu, Xiao-Jiang Li, Xiangyu Guo declare that they have no conflict interest. All institutional and national guidelines for the care and use of laboratory animals were followed.
