# Supplementary material for: Primate abnormal spindle-like microcephaly-associated knockout causes severe microcephaly and oligodendrocyte loss in the brain
Source: Protein Cell. 2025 Nov 10;17(5):471–5. doi: 10.1093/procel/pwaf097 (PMC13161476; doi:10.1093/procel/pwaf097)
Supplement: pwaf097_Supplementary_Data [file pwaf097_supplementary_data.zip › PAC-25560-LXJ-Supplementary materials.pdf]

Supplementary Materials

Methods and materials

Animals. Adult healthy cynomolgus monkeys (*Macaca fascicularis*) were housed in individual cages at Yuanxi Biotech Inc. Guangzhou and used in this study. All animal procedures were approved by the Institutional Animal Care and Use Committee at Yuanxi Biotech Inc. Guangzhou. All methods were performed in accordance with the relevant guidelines and regulations.

Cas9 vectors and gRNA. Cas9 plasmid (Addgene, #42251) was used to express spCas9 nuclease (Streptococcus pyogenes) under the control of the CMV or T7 promoter. This Cas9 carries the NLS sequences (CCCAAGAAGAAGAGGAAAGTC) at its C-terminus. The p-U6-gRNA and p-T7-gRNA expression vectors, which were used separately for HEK293 transfection and in vitro transcription of gRNAs, were provided by Dr. Liangxue Lai at The Guangzhou Institutes of Biomedicine and Health, CAS. gRNAs were designed to target exon 3 and exon 9 of the monkey *ASPM* gene (target sequence information is listed in Table S1). Template DNAs for in vitro transcription were generated by PCR amplification of the gRNAs plasmids. The PCR products were purified and transcribed by mMESSAGE mMACHINE T7 kit (Ambion, AM1344) in vitro.

Ovarian stimulation and recovery of monkey oocytes. The methods for cynomolgus monkey ovarian stimulation and oocyte recovery are similar as described previously (Tu et al., 2017; Yang et al., 2019; Tu et al., 2023). Regular cycling females aged 5–8 years were subjected to follicular stimulation using twice-daily intramuscular injections of 18 IU of recombinant human FSH (rhFSH) for 8 days; then 1000 IU of human chorionic gonadotrophin (HCG) were injected on day 9. Cumulus-oocyte complexes were isolated by surgery operation and aspiration 37 h post-rhCG. Follicular contents were placed in Hepes-buffered Tyrode's albumin lactate pyruvate medium (TALP-Hepes) containing 0.3%BSA at 37°C, supplemented with 5 IU/ml of

heparin (Sigma, Inc.). Oocytes were stripped of cumulus cells with pipetting 45–60 seconds and then filtered through a 70 μm cell strainer and collected in a 60 mm petri dish containing 5–7 ml of TALP-Hepes. Oocytes were picked up under a dissecting microscope to separate GV (intact germinal vesicle), metaphase I (GVB, no germinal vesicle, no polar body), metaphase II (MII, first polar body present), and other dead oocytes. Oocytes were rinsed and then transferred to 50 μl pre-equilibrated maturation medium that contained Connaught Medical Research Laboratories medium 1066 (CMRL-1066; Invitrogen Inc.) supplemented with 10% heat-inactivated fetal bovine serum (FBS), 40 μg/ml sodium pyruate, 150 μg/ml glutamine, 550 μg/ml calcium lactate under mineral oil. Immature oocytes such as GVB or GV cells were cultured in a 50 μl drop at 37.5°C in humidified air (6% CO^2) for up to 24h.

Male cynomolgus macaques were electro-ejaculated with a current isolation stimulator (JL-C4 V2a, JIALONG, China) equipped with electrocardiographic pad electrodes for direct penile stimulation (30–50V, 20-msec duration, 18 pulses/sec). Semen samples were collected into 15 ml tubes. Ejaculated sperm were diluted to 2×10^5 in 10% polyvinylpyrrolidone (PVP) to reduce motility and were placed in a separate drop on the manipulation dish. One single sperm was aspirated from the sperm drop into the injection needle and transferred to the oocytes TALP-Hepes drop. MII oocytes were held by holding pipet on the polar body at the 6 o'clock or 12 o'clock position, then injected by the injection needle with a sperm through the zona pellucida into cytoplasm. After ICSI, oocytes were washed twice in Hamster Embryo Culture Medium 9 (HECM-9) before being transferred into a pre-equilibrated 50 μl drop of HECM-9, covered with mineral oil and incubated at 37.5°C with 6% CO^2 for 8–10 h. Oocytes with a second polar body and two pronuclei arising after ICSI were confirmed as successful fertilization. Zygotes were injected with Cas9 mRNA and gRNAs, injected zygotes were cultured for embryo development. Embryos at 4–8 cell stages were used for transfer. Pronuclear formation was recorded 16–20 h post-ICSI, and the progression of embryo growth was recorded daily.

Cas9/sgRNA injection of one-cell embryos. The zygotes were injected with Cas9 mRNA (200 ng/μl) and gRNAs (50 ng/μl each). Microinjections into the cytoplasm of zygotes were performed as previously described (Yang et al., 2019; Tu et al., 2023) with Narishige microinjection system (Narishige Inc., Japan) under standard conditions. The zygotes were cultured in embryo culture medium-9 (HECM-9) containing 15% fetal calf serum (Hyclone Laboratories, SH30088.02) at 37.5°C in 6% CO^2. Cleaved embryos of high quality at the 4-cell stage were transferred into the oviduct of the matched recipient female monkeys. Typically, three embryos were transferred into each surrogate. The earliest pregnancy diagnosis was performed by ultrasonography about 30–35 days after the embryo transfer. Both clinical pregnancy and number of fetuses were confirmed by fetal cardiac activity and presence of a yolk sac as detected by ultrasonography.

PCR analysis of targeted sites. Tissue DNAs were extracted for genotyping by PCR. The *ASPM* genes containing the target sites were determined by PCR with primers for each one. For T7E1assay and sequence analysis of targeted exon 3 and 9, PCR primers sequences are listed in Table S2. PCR was performed by initial incubation at 95°C for 5 min, followed by 35 cycles of 95°C for 30s, 62°C for 45s, and 72°C for 45s. The PCR products were analyzed by T7E1 assay or digested by BsmAI enzymes to detect the targeted DNA mutations. PCR products corresponding to genomic modifications were then subcloned for sequencing to verify the mutation sequences.

Western blot analysis. For western blot analysis, monkey brain tissues were lysed in ice cold RIPA buffer (50 mmol/L Tris, pH 8.0, 150 mmol/L NaCl, 1 mmol/L EDTA pH 8.0, 1 mmol/L EGTA pH 8.0, 0.1%SDS, 0.5%DOC, 50 mmol/L NaF and 1%Triton X-100) containing Halt protease inhibitor cocktail (Thermo Scientific) and PMSF. The lysate was incubated on ice for 30 min, sonicated, and centrifuged at 3000 xg for 10 min. Protein density in supernatant was determined by BCA assay and then resolved by SDS-PAGE and subjected to Western blot analysis. Antibodies information is listed

in Table S3. Acquired images were subjected to densitometric quantitation using Image J software.

Immunohistochemistry and Immunofluorescence. For immunohistochemistry (IHC), monkey tissues were fixed overnight (12–16 h) in 4% PFA (paraformaldehyde) in 0.01 mol/L PBS, and then transferred into 30% sucrose at 4°C to let the brain completely sink to the bottom of the tube. Brain tissue was sectioned at 20 μm using a cryostat at −19°C. Monkey tissue slides were fixed for 10 min in 4% PFA in 0.01 mol/L PBS at room temperature, blocked with 0.1% TritonX-100/2% NGS/3% BSA/1× PBS for 30 min, and incubated with primary antibodies to related proteins in 3% BSA/2% NGS/1×PBS overnight at 4°C. Then slices were washed three times with 1×PBS and incubated with secondary antibodies at room temperature for 1 hour. Double immunofluorescence staining was analyzed using a confocal imaging system (Olympus FV3000 Microscope). Immunohistochemistry was performed using a VECTASTAIN ELITE ABC Universal Kit (Vector Laboratories, Peterborough, United Kingdom). IHC quantification analysis was performed using HALO 2.3 software (Indica Labs). The number of NeuN-positive cells for each analysis area were calculated and expressed as density (number of positive cells per mm^{-2}). Primary antibodies are listed in Table S3.

Statistical analysis. Statistical significance was assessed using the 2-tailed Student's t test for comparing two groups. When analyzing multiple groups, we used one-way ANOVA to determine statistical significance. For monkeys that were repeatedly subjected to behavioral tests, we analyzed the data using two-way ANOVA. Data presented in figures are mean±SEM. Calculations were performed with GraphPad Prism software.

References:

Tu, Z., Yan, S., Han, B., Li, C., Liang, W., Lin, Y., Ding, Y., Wei, H., Wang, L., Xu, H., *et al.* (2023). Tauopathy promotes spinal cord-dependent production of toxic amyloid-

beta in transgenic monkeys. Signal Transduct Target Ther 8, 358.

Tu, Z., Yang, W., Yan, S., Yin, A., Gao, J., Liu, X., Zheng, Y., Zheng, J., Li, Z., Yang, S., *et al.* (2017). Promoting Cas9 degradation reduces mosaic mutations in non-human primate embryos. Sci Rep 7, 42081.

Yang W, Liu Y, Tu Z, Xiao C, Yan S, Ma X, Guo X, Chen X, Yin P, Yang Z, Yang S, Jiang T, Li S, Qin C, Li XJ. CRISPR/Cas9-mediated PINK1 deletion leads to neurodegeneration in rhesus monkeys. Cell Res. 2019 Apr;29(4):334-336.

Table S1. gRNA target sequence.

gRNA	Location	Strand	Sequences
1	Exon 3	sense	GCCATGTATTCCTGAATATCAGGG
2	Exon 3	sense	GATGTCTCAACAGTGCAGTGGG
3	Exon 9	antisense	CCTTGTCTCTTCTGTAAAGATGC

Table S2. Primer sequence.

Primer	Sequences
Exon3-3F	AAGCCTGTGCATTTACAA
Exon3-3R	CTTTTCCGAGCAACTGAAGC
Exon9-9F	TGGTGCCTGCATGTAGTAGG
Exon9-9R	TGCTGGTGAAAGGTAAACCA
Exon3-3F-1	CCATGTATTCCTGAATATATCAGGG
Exon9-9F-1	TCATGATCCTTGTCTCTTCTGTA

Table S3. Antibodies used in this study

Antibody	Supplier	Species	Cat no.	Application/Dilution
ASPM	Novus Biologicals	Rabbit	NB100-2278	IHC (1:200); IF (1:500)
NeuN	Abcam	Rabbit	ab177487	IHC (1:2000)
PSD95	Santa Cruz	Rabbit	D27E11	WB (1:1000); IF (1:300)
MAP2	Abcam	Chicken	ab5392	WB (1:5000)
Synaptophysin	GeneTex	Mouse	GTX633972	WB (1:5000)
SNAP25	Abcam	Rabbit	ab41455	WB (1:2000)
Vinculin	Merck Millipore	Mouse	MAB3574	WB (1:1000)
MBP	Merck Millipore	Rabbit	MAB386	WB (1:1000)
Olig2	Abcam	Rabbit	ab109186	WB (1:2000)

Olig2	Sigma	Mouse	MABN50	IHC (1:500)
MOG	Sigma	Mouse	MAB5680	WB (1:1000)
DCX	Abcam	Rabbit	ab18723	WB (1:1000)

Supplementary figure legends

Figure S1. Gene targeting, head circumference and activity of *ASPM* KO monkey. (A) T7E1 assay of placental DNA from wild-type (WT) and *ASPM* KO monkeys, along with two aborted tissues, confirmed successful targeting of exon 3. Analysis of exon 9, which contains a native BsmAI restriction site, revealed that successful CRISPR/Cas9 targeting introduced mutations that abolished this site, resulting in resistance to BsmAI digestion. (B) The brain size of the *ASPM* KO and the age-matched control monkeys (n=12) during development. (C) The activity of *ASPM* KO and the control monkey.

Figure S2. Representative hematoxylin and eosin (H&E) stains of heart, liver, and kidney tissues are shown from both control and *ASPM* KO monkeys. The morphology of these tissues was comparable between *ASPM* KO and control monkeys, with no observable differences.

Figure S3. GFAP and Iba1 expression in *ASPM* KO and control monkey brains. (A, B) Western blotting (A) and immunocytochemistry (B) of GFAP in *ASPM* KO and control monkey brains. (C) Western blotting of IBA1 in the cortex, hippocampus, brain stem, and hypothalamus of *ASPM* KO and control monkeys. (D) IBA1 immunostaining of the white matter of *ASPM* KO and control monkeys.

Figure S4. (A) The cortical layer thickness (μm) in the *ASPM* KO monkey was reduced compared to the control. (B) Analysis of snRNAseq data from developing non-human primate brains (Han et al., Nature 2022) showing the expression of *ASPM* in mature oligodendrocytes (ODC) and oligodendrocyte precursor cells (OPC). The deposited data (https://db.cngb.org/nhpca/download) were analyzed using Seurat package (v4.2.0)[111] within an R environment (v4.1.3).

Figure S5. *ASPM* isoforms and gene targeting in different models. (A) Human *ASPM* is expressed as a full-length transcript and three isoforms generated by alternative splicing. Isoform 1 lacks exon 18, and both isoform 2 and 3 lack exons 4–17, producing a large truncation. Additionally, Isoform 2 also lacks exon 27. Mouse *Aspm* is expressed as a full-length transcript and one major isoform (Isoform 1), which lacks exon 18, similar to human Isoform 1 （Kouprina et al., 2005）(B) Gene targeting strategies have been used to disrupt *ASPM* in different species. In primates, deletion of exons 3–10 is predicted to abolish the expression of all functional *ASPM* isoforms due to the removal of critical coding regions in all these isoforms, resulting in severe microcephaly phenotypes and oligodendrocyte loss.

Supplementary video: Reduced size of head and body and decreased activity of

ASPM KO monkey as compared with the control monkey. The animals were at the age of 5 months.

Fig. S1

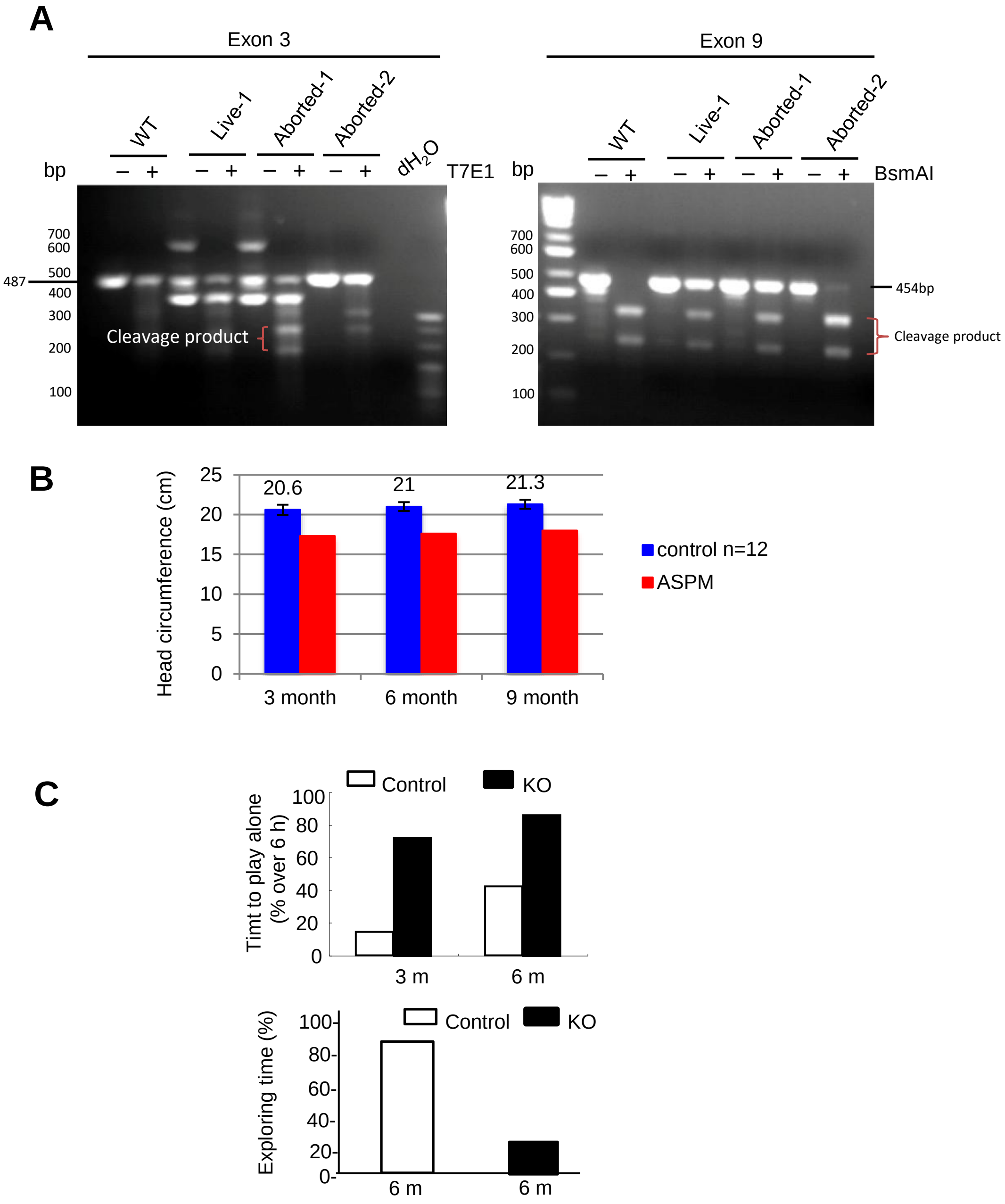
A
Exon 3
WT
Live-1
Aborted-1
Aborted-2
dH2O
T7E1
bp
700
600
500
487
400
300
200
100
Cleavage product
Exon 9
BsmAI
454bp
Cleavage product
B
Head circumference (cm)
25
20
15
10
5
0
20.6
21
21.3
3 month
6 month
9 month
control n=12
ASPM
C
Control
KO
Timt to play alone
(% over 6 h)
100
80
60
40
20
0
3 m
6 m
Exploring time (%)
6 m
6 m

Fig. S2

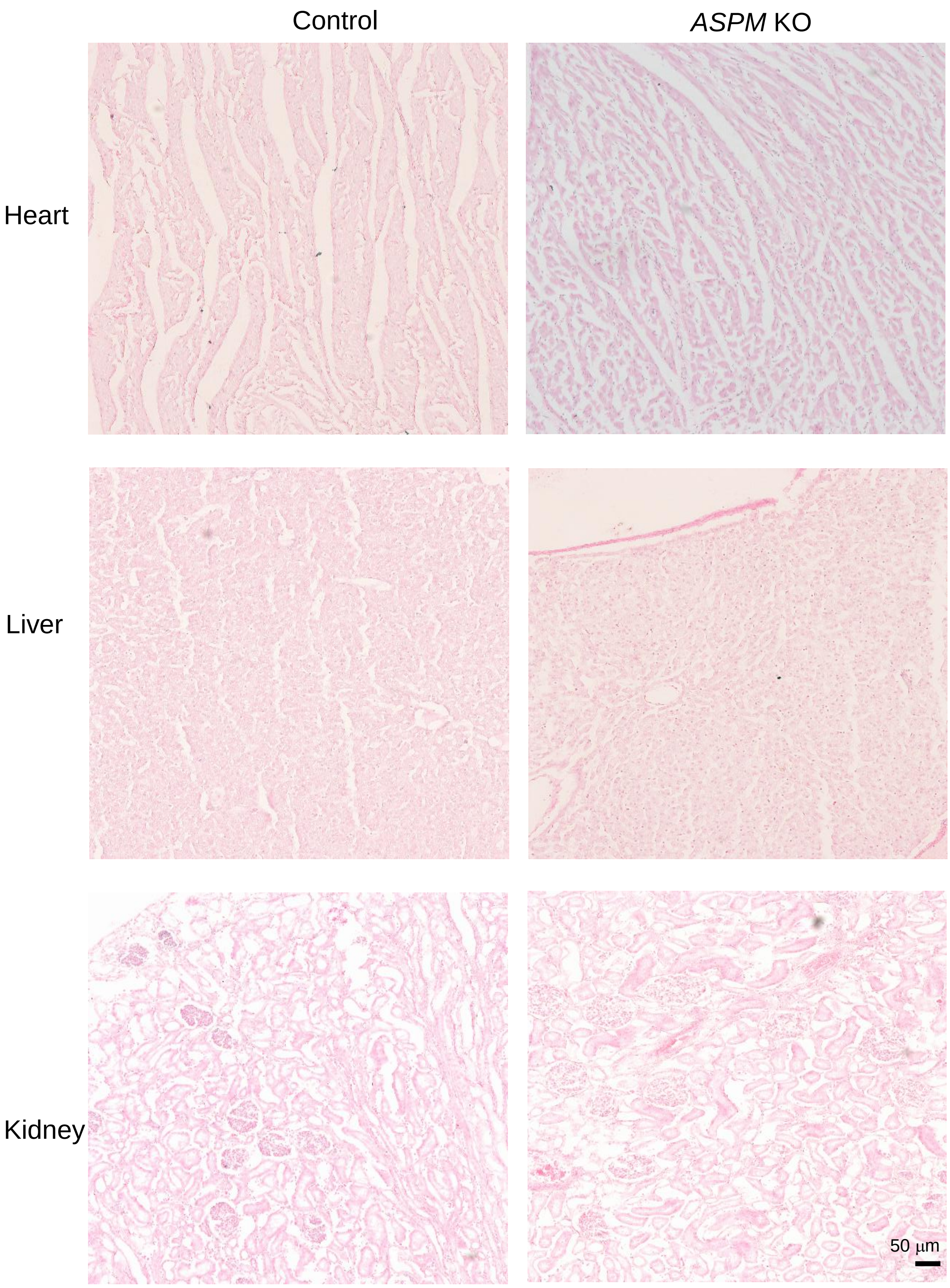

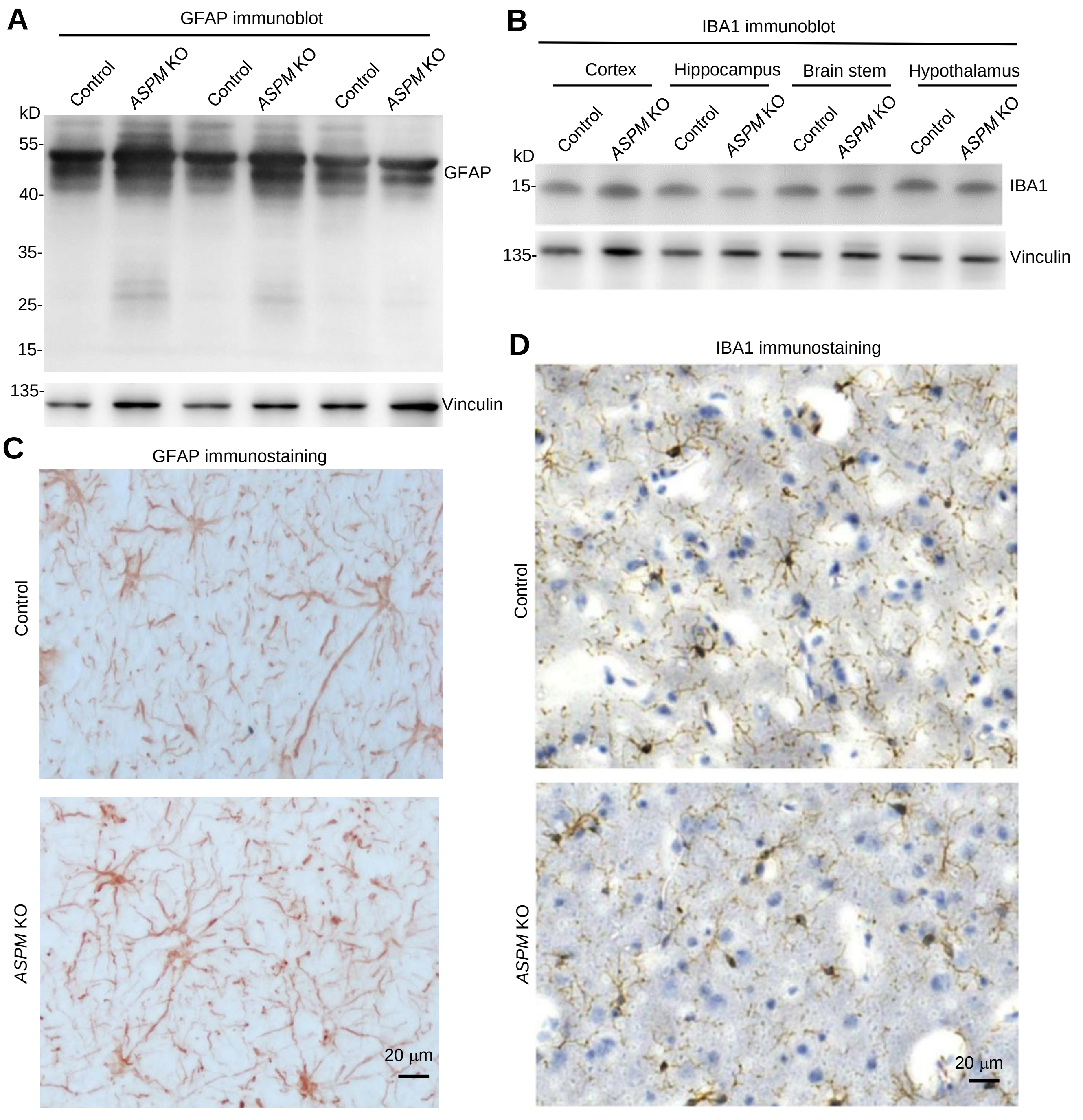
A
GFAP immunoblot
Control
ASPM KO
Control
ASPM KO
Control
ASPM KO
kD
55-
40-
35-
25-
15-
135-
GFAP
Vinculin
B
IBA1 immunoblot
Cortex
Hippocampus
Brain stem
Hypothalamus
Control
ASPM KO
Control
ASPM KO
Control
ASPM KO
Control
ASPM KO
kD
15-
135-
IBA1
Vinculin
C
GFAP immunostaining
Control
ASPM KO
20 μm
D
IBA1 immunostaining
Control
ASPM KO
20 μm

Fig. S4

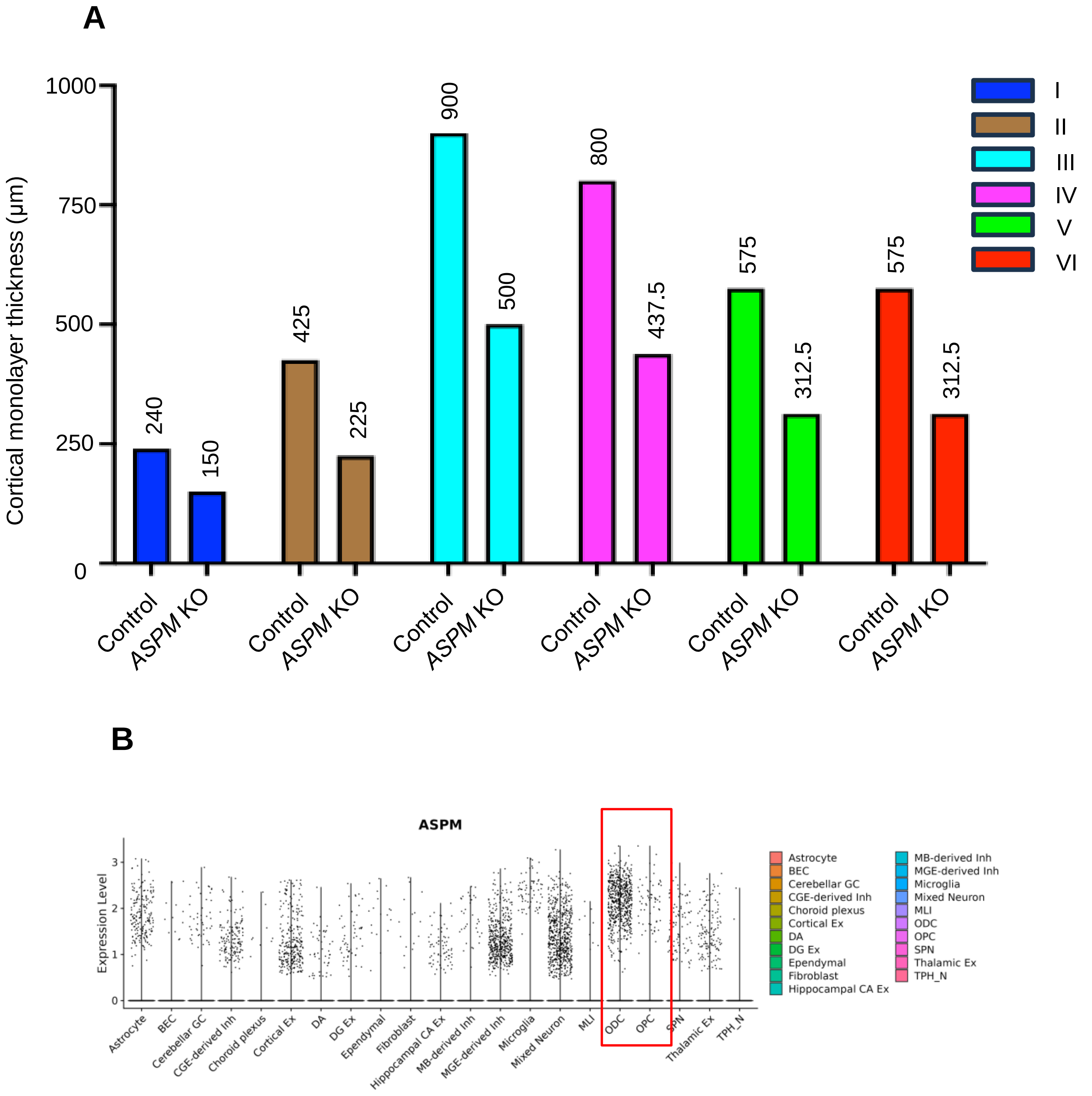
A
Cortical monolayer thickness (μm)
1000
750
500
250
0
240
150
425
225
900
500
800
437.5
575
312.5
575
312.5
Control
ASPM KO
I
II
III
IV
V
VI
B
ASPM
Expression Level
Astrocyte
BEC
Cerebellar GC
CGE-derived Inh
Choroid plexus
Cortical Ex
DA
DG Ex
Ependymal
Fibroblast
Hippocampal CA Ex
MB-derived Inh
MGE-derived Inh
Microglia
Mixed Neuron
MLI
ODC
OPC
SPN
Thalamic Ex
TPH_N

Fig. S5

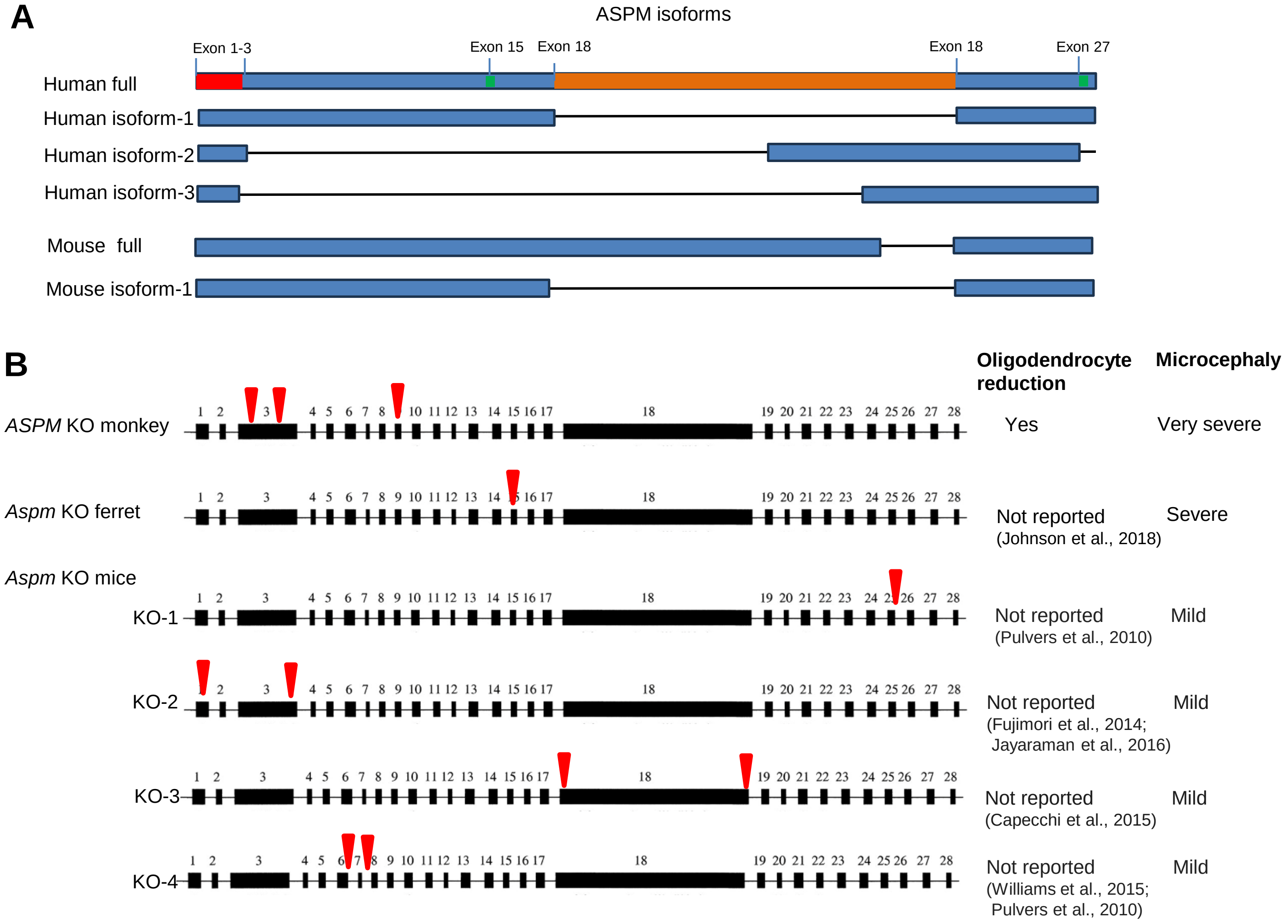
A
ASPM isoforms
Exon 1-3
Exon 15
Exon 18
Exon 18
Exon 27
Human full
Human isoform-1
Human isoform-2
Human isoform-3
Mouse full
Mouse isoform-1
B
Oligodendrocyte reduction
Microcephaly
ASPM KO monkey
Yes
Very severe
Aspm KO ferret
Not reported (Johnson et al., 2018)
Severe
Aspm KO mice
KO-1
Not reported (Pulvers et al., 2010)
Mild
KO-2
Not reported (Fujimori et al., 2014; Jayaraman et al., 2016)
Mild
KO-3
Not reported (Capecchi et al., 2015)
Mild
KO-4
Not reported (Williams et al., 2015; Pulvers et al., 2010)
Mild
1 2 3 4 5 6 7 8 9 10 11 12 13 14 15 16 17 18 19 20 21 22 23 24 25 26 27 28
